# Computational analysis and determination of a highly conserved surface exposed segment in H5N1 avian flu and H1N1 swine flu neuraminidase

**DOI:** 10.1186/1472-6807-10-6

**Published:** 2010-02-22

**Authors:** Ambarnil Ghosh, Ashesh Nandy, Papiya Nandy

**Affiliations:** 1 Physics Department, Jadavpur University, Jadavpur, Kolkata 700032, India; 2 School of Environmental Studies, Jadavpur University, Jadavpur, Kolkata 700032, India; 3 Centre for Interdisciplinary Research and Education, Jodhpur Park, Kolkata 700068, India

## Abstract

**Background:**

Catalytic activity of influenza neuraminidase (NA) facilitates elution of progeny virions from infected cells and prevents their self-aggregation mediated by the catalytic site located in the body region. Research on the active site of the molecule has led to development of effective inhibitors like oseltamivir, zanamivir etc, but the high rate of mutation and interspecies reassortment in viral sequences and the recent reports of oseltamivir resistant strains underlines the importance of determining additional target sites for developing future antiviral compounds. In a recent computational study of 173 H5N1 NA gene sequences we had identified a 50-base highly conserved region in 3'-terminal end of the NA gene.

**Results:**

We extend the graphical and numerical analyses to a larger number of H5N1 NA sequences (514) and H1N1 swine flu sequences (425) accessed from GenBank. We use a 2D graphical representation model for the gene sequences and a Graphical Sliding Window Method (GSWM) for protein sequences scanning the sequences as a block of 16 amino acids at a time. Using a protein sequence descriptor defined in our model, the protein sliding scan method allowed us to compare the different strains for block level variability, which showed significant statistical correlation to average solvent accessibility of the residue blocks; single amino acid position variability results in no correlation, indicating the impact of stretch variability in chemical environment. Close to the C-terminal end the GSWM showed less descriptor-variability with increased average solvent accessibility (ASA) that is also supported by conserved predicted secondary structure of 3' terminal RNA and visual evidence from 3D crystallographic structure.

**Conclusion:**

The identified terminal segment, strongly conserved in both RNA and protein sequences, is especially significant as it is surface exposed and structural chemistry reveals the probable role of this stretch in tetrameric stabilization. It could also participate in other biological processes associated with conserved surface residues. A RNA double hairpin secondary structure found in this segment in a majority of the H5N1 strains also supports this observation. In this paper we propose this conserved region as a probable site for designing inhibitors for broad-spectrum pandemic control of flu viruses with similar NA structure.

## Background

A pandemic occurs when a new viral strain appears, against which the human population has no immunity, resulting in epidemics worldwide with high mortality and morbidity. It is estimated that the influenza pandemic that started with the 1918 Spanish flu killed ~20 to 50 million people worldwide [1], followed by epidemics of Asian flu in 1957, Hong Kong flu in 1968 and Russian flu in 1977, each with random severe attacks on human populations [2]. A recent strain of influenza, the highly pathogenic avian influenza (HPAI) H5N1, and its variants have been in circulation since the first major outbreak in 1997 among birds in South East Asia leading to 141 human deaths [3]. High mutation rate and wide variety of birds and mammals including human hosts are probable reasons of pandemic-causing ability of the virus. Previous studies on the pathogenicity of influenza virus have reported the role of different kinds of genetic events like antigenic shift, antigenic drift, recombination and reassortment as major reasons for the emergence of virulent strains [4-10]. The very recent outbreak of swine influenza (H1N1) in Mexico in April/May 2009 has already prompted the WHO raise an alarm at the situation by raising the level of influenza pandemic alert to phase 6 [11]; the H1N1 swine flu is believed to be a product of reassortment between genes in the avian, human and swine influenza strains which has exhibited capability for human to human transmission and resulted in at least 8768 human deaths worldwide at last count [11].

According to the latest WHO report (from 2003 to 27^th ^November 2009), 262 out of 444 H5N1 flu infected human patients have died [12]. Although there is no confirmed evidence of human to human transmission, WHO still considers the H5N1 to be a potential pandemic threat [12]. The only known and effective inhibitors developed to date to control the spread of this virus are targeted against either the M protein (amantadine, rimantadine) or NA (oseltamivir (marketed as Tamiflu), zanamivir (marketed as Relenza)). Amantadine and rimantadine resistance has developed in almost all circulating influenza strains [13] and therefore only oseltamivir and zanamivir are currently being stockpiled as precaution against any pandemic [12]. However, though these drugs are effective against the NA active site, recent strains from localized areas [Northern Hemisphere (2007-08 season), Southern Hemisphere (2008 season) and finally 100% resistant in United States (2009 pandemic strains)] have developed resistance against oseltamivir [14]. Oseltamivir treatment showed resistance in up to 2% patients in clinical trials and 18% of treated children including frequent resistance acquisition in case of children only [15,16]. A few Tamiflu resistant cases were also reported in laboratory experiments [14,17], where the mutation of H274Y is believed to be partly responsible [18]. Additionally, in case of zanamivir, markedly reduced effectiveness (2.3% of collected sample) was observed in influenza-A (H1N1) viruses isolated between 2006 to early 2008 from Australia & Southeast Asia containing previously undescribed Q136K NA mutation [19]. Thus, the high rate of mutation in the viral sequences has always posed a risk of rapid development of resistance against current inhibitors and vaccines. Detection of any strongly conserved region within the overall mutational scenario remains therefore an important point of focus for designing effective remedies covering broad spectrum antiviral activity.

In a previous analysis [20] using graphical representation methods, we had reported a specific stretch in the 3' C-terminal end of the RNA sequence that seemed to be well conserved. Graphical representation techniques were developed by Hamori and Ruskin [21], Gates [22], Nandy [23] and Leong and Morgenthaler [24], among others [25] and have been applied to a wide variety of problems highlighting their usefulness. Liao et al [26] have shown that such techniques can be used to analyse the SARS corona virus, and, separately [27], to generate phylogenetic trees without any need for multiple alignments, Larionov et. al. [28] have shown that plots of human and mouse chromosomal sequences in a graphical representation were able to reveal long range palindromes. Randic, Humberto Gonzales-Diaz and several other authors have extended these techniques to protein sequence analysis and obtained many useful results. Parameters like numerical indices, topological indices are the recent outcome of these techniques applied to proteins, viral surfaces, RNA secondary structures and small molecules [29-34] have extended the scope to consider more general biological applications. In particular, González-Díaz et al. extended these representations to the study of protein sequences [35] and Mass Spectra outcomes of proteins and/or protein serum profiles in parasites [36], Toxicoproteomics and diagnosis of Cancer patients [37,38]. Also, these descriptors can be used in QSAR studies of biological entity in molecular level. These QSAR connect structural information with the biological function of a molecular entity under study and may be used to predict unknown entries. Structure here refers not only to drug structure but also to DNA sequence, RNA sequence or secondary structure, and protein sequences or 3D structure [38]. Analyses based on graphical representation techniques have thus become acceptable for many purposes [39].

In this study we have used the 2D graphical representation model for gene sequences [23] and a 20D graphical representation method for proteins [40] to analyze a database consisting of 514 NA sequences of H5N1 and 425 sequences of the H1N1 influenza subtypes. In the protein algorithm we modelled a protein sequence in the abstract using a 20-dimensional Cartesian coordinate system to generate sequence descriptors. While we lose the benefits of visual recognition, our method allows easy mathematical closure and comparison of characteristic numbers to determine the degree of relatedness of or patterns in different sequences and peptide stretches.

Furthermore, numerical characterisation techniques based on graphical representations have enabled quantitative estimation of sequence similarities and dissimilarities [25]. Basically there are two approaches for numerical characterization, both of which use the graphical representation to map a DNA sequence into a set of numbers. One approach using geometrical mapping proposed by Raychaudhury and Nandy [41] have been found to be useful for several calculations based on the 2D graphical representation [25], and extended recently to an abstract 20D modelling for protein sequences [40], where individual sequences are indexed by numerical descriptors. The other approach is to use matrix methods by forming ratios of graph theoretic and Euclidean distances between nodes of the graphical plots, first formulated for DNA sequences in Randic et al [42]. Since invariants associated with matrix formulation are well-known, individual sequences can be indexed by one or more such invariants of various orders; it is expected that these would be sufficiently characteristic of the underlying sequences to enable unique characterization. This technique has been the most widely used method of choice for the researchers in this field who have defined different types of matrices to construct various invariants to describe the DNA sequences. However, the difficulties associated with computing various parameters for very large matrices that are natural for large sequences have restricted the numerical characterizations to leading eigenvalues and the like [25].

In the current work with emphasis on the protein sequence, the body region of the NA was considered for deeper analysis due to its role in docking and its large surface exposed segments along with considerable variability throughout the sequence. The body segment also holds the active site where sialidase activity takes place, while the C-terminal end of a membrane protein is also very important since it's generally responsible for holding information of protein transport [43], folding stabilization [44], cell to cell migration specificity [45] and others. One related example is the C-terminal of ryanodine receptor (RyR) channel: The 100 amino acids situated at C-terminal of RyR, referred to as the C-terminal tail, is a highly conserved sequence throughout RyR isoforms and which has been implicated in channel function where deletion of final 15 residues results in an inactive channel [46]. Additionally Goto et al. [47] have shown the importance of carboxy-terminal lysine (position 453) of NA in plasminogen mediated hemagglutinin cleavage. Li et al has shown from the DNA sequences that truncation of 66 nucleotides at the 3'-terminal (or C-terminal 22 amino acids) of the NA leads to loss of antigenicity against influenza virus in BALB/c mice [48], indicating a crucial role of that region as a potent antigen. Additionally, we have modelled the RNA secondary structure of this region and determined that a double loop hairpin formation exists in a majority of the sequences. All these evidences support the importance of C-terminal region as a functional and antigenically important portion for the NA proteins.

## Methods

We selected the complete cds of the H5N1 NA gene sequences for the period 1997 to 2007, totalling to 514 samples, available in the GenBank DNA database [as on March 10, 2008]. They comprised 35 sequences with 1410 bases (469 translated aa), 8 sequences with 1353 bases (450 aa) and 471 sequences of 1350 bases (449 aa). Of the total 471 strains of the 1350 base NAs that are more widely prevalent now, 371 strains were from avian, 96 were from human isolates and 4 from other organisms. The 1410 base NAs comprised of a single human isolate and the rest are avian; there were no human isolates having 1353 nucleotides. To complement the study of H5N1 we also selected 425 complete cds's of the H1N1 swine flu NA sequences deposited in the GenBank [up to 23^rd ^July 2009].

### Graphical representation method for nucleic acids

In the Nandy 2D graphical representation method [23] a nucleotide sequence is plotted on a 2D Cartesian axes system as follows: Move one step in the negative x-direction for an adenine (a) in the sequence, one step in the positive y-direction for a cytosine (c), one step in the positive x-direction for a guanine (g) or one step in the negative y-direction for a thymine (t) to plot a point. Start from this point and do the same exercise for the next base in the sequence and so on, which ultimately generates a graph of the sequence as a series of points on the plot essentially representing the composition and distribution of bases in the sequence.

To make quantitative comparisons between different sequences plotted in this system, we follow the method given in Raychaudhry and Nandy [41]. The weighted centre of mass of each graph is calculated by summing the co-ordinate values of each point and then a graph radius, g_R_, is computed. The g_R _represents the Base Distribution index or numerical descriptor for nucleic acids. The g_R _is a very sensitive measure of the sequence composition and distribution [41,49], the values depending on the type of mutations and where in the sequence they occur. g_R _is especially useful in comparing equal length sequences [50].

### Graphical representation of proteins

To characterize protein sequences graphically, we use a recently proposed novel method where we model a protein sequence in the abstract using a 20-dimensional Cartesian coordinate system which has been shown to be a useful technique for sequence comparisons and phylogenetic studies [40]. In this method we associate each amino acid with one axis of a 20D Cartesian coordinate system; the choice of association is equivalent for all residues, but once assigned will be fixed for the duration of the computation. For easy computation and comparison we have calculated weighted averages and resultant vectors that are unique to the respective sequences as in the case of the nucleotide sequence representations [40]. Here this technique is used to compute the protein descriptors to determine similarity/dissimilarity between different protein sequences including short protein stretches. To avoid error of false similarity when analysing short sequences, we have added a stretch of 20 aa peptides at the beginning of each sequence for filling up all the initial coordinates with the value 1 before starting the calculation of protein descriptors, a technique that is useful for comparative studies.

### RNA Secondary Structure Prediction Method

**Figure 1:**
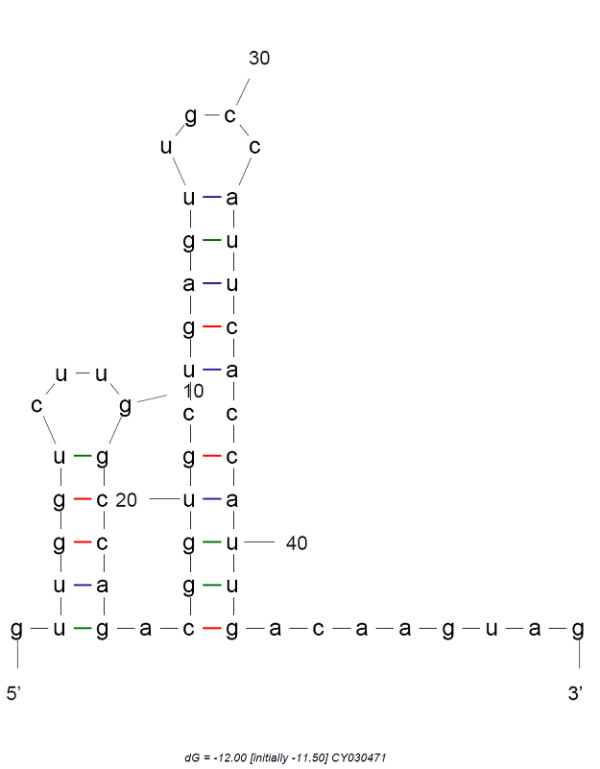
**Double hairpin forming conserved RNA structure from 3' terminal**. Double hairpin structure from RNA secondary structure prediction server of 3'-terminal 50 base region. The strain used for here is A/duck/Laos/NCVD-35/2007(H5N1) and the g_R _value of this sequence is most widely distributed among the whole database.

To predict RNA secondary structure from the conserved 3'-terminal RNA sequence we have used the mfold RNA secondary structure prediction server (version 3.2) [51] of Zuker and Turner [52,53] (Figure 1).

### Graphical Sliding Window Method (GSWM)

**Figure 2:**
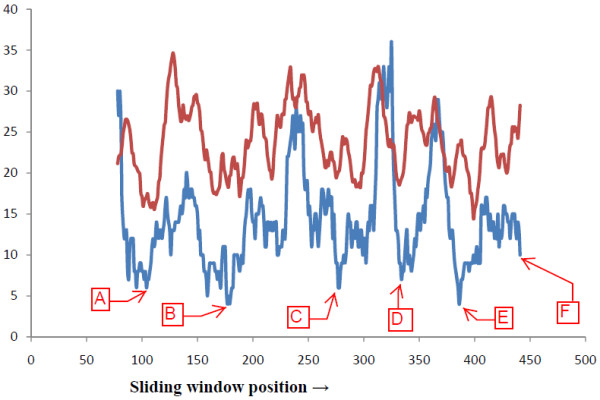
**Comparative graphical representation of aa segment variability and ASA**. In this graph Average Solvent Accessibility (ASA) (in brown) is compared with GSWM generated amino acid segment variability (in blue). The y-axis represents both the variability and solvent accessibility. The x-axis represents the sliding window middle position number.

To determine the relative degree of variability of this C-terminal 16 aa stretch, we subjected the entire body region to a scan of 16 aa stretches by sliding a 16 aa wide window residue by residue on the 20D graph and calculating the protein descriptor p_R _at each point for all 514 H5N1 protein sequences. From this dataset we determined how many p_R_'s were different; the lower the number the lower the variability of the stretch. We also determined the solvent accessibility at each point by using the SABLE solvent accessibility prediction server on the Internet [54]. The results of this Graphical Sliding Window Method (GSWM) were then compared (Figure 2) with the 16-residue moving average solvent accessibility data to determine correlations, if any, and conserved regions with considerable solvent accessibility.

**Figure 3:**
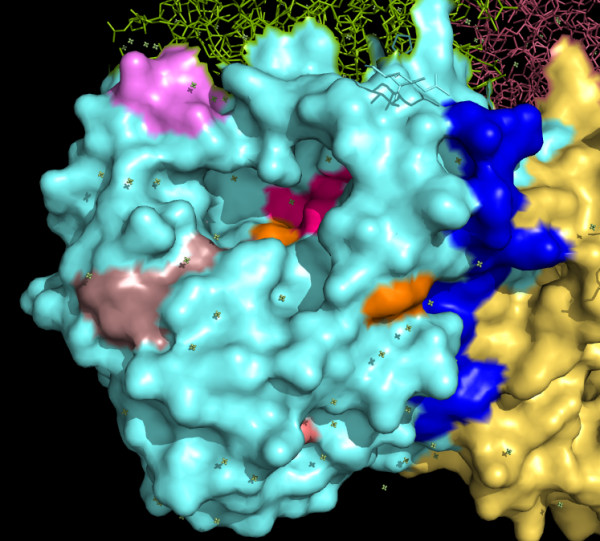
**Distribution of conserved sequence stretches on neuraminidase surface**. Surface penetration of portions of the highly conserved sequences determined from Figure 2. Last 16 aa region corresponding to point F of Figure 2 is coloured blue. 16-aa segment corresponding to point A in Figure 2 is shown here in cherry red colour, that of point B in pink, point C in dark chocolate, point D in deep salmon and point E in orange. These are large stretches of which only parts are visible on the surface, but much lesser in extent than the last 16 aa stretch corresponding to point F of Figure 2.

The choice of the 16 aa block of residues for this exercise was dictated by, initially, the observation of the 50-base conserved nucleotide stretch at the C-terminal end of the gene sequence, and further supported by the following: (a) We started with a 8-residue block since this is the consensus minimum number for identifying a protein from a single block and performed a GSWM analysis. This was then extended to 12-residue, 16-residue and 24-residue blocks, based on which the 16-residue block was found to yield optimum results. (b) A second consideration was that while a short aa segment may show a high probability of being solvent exposed, a comparatively large segment will have considerable portions embedded in the protein. (c) A large segment will have considerable variability in the amino acid composition. Indeed, based on these considerations, the GSWM identified the 16 aa segment as the optimum and this can be seen in the figures (Figure 3) and the accompanying animated sequence [Additional file 1] where it will be observed that all but one of the six 16-aa blocks show only portions as surface exposed.

### Average Relative Solvent Accessibility prediction

To analyze solvent accessibility we have used SABLE secondary structure prediction server [54]. Body regions of twenty proteins were submitted to calculate relative solvent accessibility.

### 3D structure Visualization

To visualize the protein structural residues and their chemical environment we had used the crystallographic structure from PDB database submitted by Russell et al. [55] and the molecular visualization tool PyMOL [56].

## Results and Discussion

To determine a potentially conserved region in the H5N1 avian flu and H1N1 swine flu NA gene and protein sequences we proceeded initially from the 2D graphical representations of the various strains of the H5N1 and H1N1 NA which sequence segment seemed to be most strongly conserved, followed by confirmation by the RNA and protein descriptors. Next, we did a sliding window analysis along the NA body region to determine whether any other segment had comparable characteristics. Finally, we did a solvent accessibility analysis of the protein. From correlation of the results obtained from solvent accessibility analysis and sliding window method we could determine that the terminal segment was the best surface exposed conserved 16 amino acids long stretch which may be involved in significant biological functions; this is especially significant in view of the observation that truncation of the last 22 aa leads to loss of biological function of NA [48]. We followed this up with a detailed study of the crystallographic features to observe that indeed this segment of the NA protein is important for its stability.

Our earlier work with the 2D graphical representation of 173 RNA sequences of the H5N1 NA had identified a 50-base segment at the 3' end of the sequence as being well conserved. NCBI-BLAST analysis had confirmed that this indeed was strongly conserved not only among the H5N1 NA but also among the NAs of influenza A subtypes H1N1, H3N1, H4N1, H6N1, H7N1, H9N1, H10N1, H11N1, and H12N1 [20]. In the current extended database of 514 H5N1 strains also we found from the graph radius descriptor g_R _that only 25 strains of the 50-base segment had any differences in the bases, i.e. 489 strains (95.14%) had this segment identical to one or the other of the 25 strains out of the 514 samples. Further, extension of the same study to samples from all nine antigenic subtypes (N1 to N9) showed considerable conserved characteristics for this protein region. A multiple alignment analysis (Table 1) of the last 20 amino acid residues (4 more residues are taken to facilitate the alignment process) of each subtype results in at least 8 well conserved column positions indicating the importance of the finding.

**Table 1:** Multiple alignment result for protein sequence of a potential conserved region (C-terminal) of last 20 amino acids of NA antigenic variants (N1 to N9).

LOCUS NAME & SUBTYPE OF NA	SOURCE & HOST ORGANISM	LAST 20 AMINO ACIDS	NUMBER OF AMINO ACIDS
EU880344 & NN6	A/mallard/Yan chen/2005(H4N6)	**SKERLGSWSWHDGAEIIYFK---**	470

AB292782 & NN9	A/duck/Hong Kong/562/1979(H10N9)	**STEFLGQWNWPDGAKIEYFL---**	470

AB450450 & NN7	A/duck/Taiwan/4201/99(H7N7)	**SPFPVGSGSFPDGAQIQYFS---**	471

AJ307613 & NN2	A/human/Montreal/MTL20/00(H3N2)	**SGT-YGTGSWPDGADINLMPI--**	469

AM933234 & NN3	A/Perdix perdix/Germany/R44/06(H8N3)	**DNE-PGSGNWPDGSNIGFMPK--**	469

ABP52008 & NN1	A/Viet Nam/1203/2004(H5N1)	**--SDTVGWSWPDGAELPFTIDK-**	449

EU429780 & NN4	A/duck/Eastern China/01/2005(H8N4)	**--SDTTGWSWPDGALLPFDIDK-**	470

AB289332 & NN8	A/duck/Hong Kong/438/1977(H4N8)	**---EVPEWSWDDGAILPFDIDKM**	471

AB270594 & NN5	A/duck/Hokkaido/1058/01(H4N5)	**---EVADWSWHDGAILPFDIDKM**	472

In comparison, in the transmembrane and stalk regions the percentage of variants, i.e. non-identical sequences, among the 514 H5N1 strains are 24.71% and 33.85%, respectively; the complete body region, i.e. from the end of the stalk to the end of the sequence, shows 75.5% variants, very high compared to the 4.86% of the 50-base 3'-terminal region. As can be expected, a large number of the mutations responsible for the RNA sequence variations in this 50-base region are synonymous. A parallel study using the protein graphical representation and the protein graph radius descriptor, p_R_, shows that the number and percentage of variants of the corresponding protein sequence are 10% and 1.95%, respectively.

We also analyzed the 425 strains of the H1N1 swine flu NA sequences to determine the degree of conservation in the 50-base segment at the 3'-end of the gene sequences and the 16 aa segment at the C-terminal end of the protein sequences. While the gene sequences are found to have mutated considerably compared to the earlier H1N1 and H5N1 strains, the mutations are all synonymous and the protein sequences are all identical with one another and to one of the dominant strains in the H5N1 sequences.

Consequent to these observations regarding the stability of this segment, we considered their predicted secondary structures. Terminal structures of viral RNAs are found frequently to be involved in host dependent cellular processes including replication, transcription, viral particle packaging, by forming secondary structures like hairpin, double-hairpin, pan-handle etc [57-59]. Numerical characterization of the 514 NA RNA database have already summarized the database to 25 strains generating 25 unique numbers each representing unique structure of the 50 base RNA segment. Using these strains in RNA secondary structure prediction through mfold have yielded a double hairpin structure (Figure 1) in 21 cases with acceptable ΔG values showing strong stability characteristics. This kind of double hairpin structures are found to contribute in some biological functions like V(D)J recombination [60], RNA editing [61] and viral RNA encapsidation [62]. This prediction for the RNA segment in H5N1 not only supports the observation of stability of this C-terminal region, but also designates the importance of that region in viral cell biology at RNA level.

To determine whether the translation product of such a segment is unique in the H5N1 NA, we obtained the protein graph descriptor of an equivalent size moving along the entire body sequence. A 16-residue window was then used in our GSWM technique as described previously. To obviate problems and degeneracies occurring in the computation of the p_R _due to the absence of one or more residues in the 16-base stretch, we added a 20-residue peptide consisting of all 20 individual amino acids to the beginning of the window and thus had a 36-residue window for the GSWM technique: The first 20 aa remains constant for all computations and the last 16 residues varied depending on the sequence. Since we were interested in determining whether and which p_R_'s were equal between strains, and not on the magnitude of the p_R_'s, this artifice proved adequate for our purpose.

Since the body region is composed of 379 aa, the GSWM gave us 379-15 = 364 p_R _values for each strain. Comparing the results positionwise between the 514 strains, we were able to determine at each position how many different p_R _were there, thus giving us a quantitative estimate of the variability in the 16-residue profile at each position for all the sequences (Figure 2). Figure 3 and Additional file 1 show that in 6 regions (marked as A to F in Figure 2) along the NA protein body primary sequence the variability is significantly low. These are specifically at around residue position numbers 104 (point A in Figure 2), 177 (B), 277 (C), 334 (D) and 386 (E) with a comparative minimum also at window position 441 (F), which corresponds to the 50-base conserved region at the 3' end of the RNA sequence.

All the 16 residue long amino acid segments other than the last region mentioned above seem better conserved than the C-terminal end segment. A potentially conserved portion in the protein needs to be solvent accessible for showing optimum interaction with its environment. However biological importance of a conserved peptide stretch increases many-fold as it becomes surface exposed or solvent accessible. Assuming that solvent accessibility may be indicative of accessibility of the site to potentially interacting portion of molecules, we next determined the solvent accessibility at each position of the sequence using SABLE secondary structure prediction server. Figure 2 (brown coloured plot) shows the results for a sample set of 20 strains used for the purpose.

To make a comparison between the variability results obtained from the GSWM for the 16-residue window analysis of the 514 H5N1 NAs, we constructed a 16-residue moving average of the solvent accessibility data. Figure 2 shows the plots of the two data sets - brown plot for the ASA and blue coloured plot for the GSWM results. A reasonably good correlation, 0.49 at 0.01 significance level, can be seen between the variability of the N1 strain protein sequences and solvent accessibility within the body region, though sequences included in stalk and transmembrane region do not show such a good correlation. We notice that regions of high solvent accessibility have high variability of the residues. This indicates that regions of the protein that are not accessible to the outside are more restricted in their mutations, whereas segments that are more easily accessible to outside influences can accommodate a greater degree of change implying that the interior of the protein is more stable, perhaps for structural reasons. This analysis shows that whatever individual hydrophobic or hydrophilic properties each residue may have, the co-operative effect of a stretch of peptides is quite important.

The last segment of the protein sequence is slightly different from the other apparently stable segments identified hitherto. In this segment, as can be seen from Figure 2, while the protein sequence variability is seen to decline, the solvent accessibility factor is seen to increase. Thus as we come closer to the last 16 residues, we find that this stretch is considerably solvent accessible, while at the same time being highly conserved. To understand this apparent divergence, we studied the crystallographic evidence of the structure of this region. Figure 3 and Additional file 1 show that this 16-residue C-terminal region of the protein sequence resides on the surface of the quaternary structure and appears to participate in the binding of one subunit of the quaternary structure to the next. It is therefore not surprising that this segment of the protein is quite well conserved for its structural stability, and it is at the same time accessible to the outside. According to recent structural biology research, a stretch of conserved surface residues or regions of a solvent accessible protein can be of significant biological importance in terms of enabling protein-protein binding [63], allosteric regulation [64], oligomerization [65], signal peptides activity [43] etc. Thus any molecule that targets this conserved surface stretch can interfere with the protein's normal biological activity leading to the protein's malfunction. Recent pharmaceutical research frequently uses allosteric modulator [66] for designing drug molecules without side effects and toxic effects. In contrast, as can be seen from Additional file 1, the other five regions that would appear from Figure 2 to be highly conserved have a majority of the sequence in the interior of the protein and thus not as accessible to the environment. Here we propose that the conserved 16 aa region at the C-terminal end as determined by our analyses can be targeted to explore it's biological importance and consequent development of inhibitory molecules targeting this section for efficient control of viral infection.

## Conclusions

Our analyses of the 16 aa stretch at the C-terminal of the NA protein for the H5N1 avian flu strains and the very recent H1N1 swine flu strains, and also other influenza subtypes, have shown its strongly conserved nature. This can be attributed to its possible role as a stitching agent for the stability of its tetrameric structure; while the gene sequence undergoes several mutations, these are mostly synonymous permitting very little variance in the amino acid composition implying that the asynonymous mutations must be eliminated due to functional requirements. Also, protein-protein interactions in such regions are known to lead to functional instability through realignment of the docking region. Comparisons with the solvent accessibility profile and 3D structure have shown that a major part of this stretch of the protein sequence is surface situated. Thus, it is possible to hypothesize that this segment is important for the stability of the NA protein and any destabilization initiated through it could lead to neutralization of the NA's effectiveness as an agent for the proliferation of the influenza virions in vivo.

## Abbreviations

NA: Neuraminidase; aa: Amino acid; C-terminal: Carboxy terminal; QSAR: Quantitative Structure Activity Relationship; GSWM: Graphical Sliding Window Method; ASA: Average Solvent Accessibility.

## Authors' contributions

All authors participated in study design, research and manuscript preparation. All authors read and approved the final manuscript.

## Supplementary Material

Additional file 1 **PyMOL generated movie showing distribution of surface exposed portions of conserved segments in NA**. The movie was generated by rocking the cyan coloured monomer in x-axis by 180 degrees. Colour schemes of the conserved segment stretches are same as in Figure 3. Five of the conserved segments are seen to have small sections surface exposed, while the 16 aa C-terminal amino acids (in blue) is found to be fully exposed and highly solvent accessible. Click here for file
